# How to Take the Temperature

**Published:** 1903-04-04

**Authors:** 


### HOW TO TAKE THE TEMPERATURE.

A somewhat extensive investigation has been recently accomplished by Dr. F. W. Burton-Fanning and Dr. S. Gurney Champion [1] to discover (1) the relationship of the readings of the thermometer according as it is placed in the mouth, the rectum, the urine, the axilla, or the groin; and (2) the effects on the temperature of exercise in health, in tuberculosis, and in some other diseases. With regard to taking the oral temperature they find that the time commonly allowed for the thermometer to remain in the mouth is too short to give a correct reading. In certain conditions as long a time as 30 minutes may be required in order to obtain a right result. This is not due to any defect in the instrument used, but represents the time taken for the buccal cavity to recover its normal heat, after it has been cooled down by a cold external atmosphere or by breathing with the mouth open. Exercise is a special source of fallacy in taking the temperature by the mouth, as it entails more rapid respiration, usually with the mouth open, with the result that the buccal cavity becomes cooled below the temperature of the rest of the body. Exercise produces a rise in temperature as demonstrated by rectal observation, but this rise is only of short duration, and does not persist beyond about half an hour from the cessation of activity. For these reasons the fact that exercise causes a rise of temperature is overlooked if the reading be taken from the mouth. Much the same effect is produced in cooling the buccal cavity by applying ice to the cheeks as is caused by rapid respiration. An ice-bag applied to the side of the face for 15 minutes reduced the temperature on that side of the oral cavity by 4·4°, after the ice-bag had been taken away it took one hour for the mouth to regain its normal temperature. In the rectum the maximum reading is obtained in from one to five minutes, and the rectal temperature is therefore a much surer guide to the true condition of affairs. The inguinal, axillary, and urinary temperatures are not such good indicators as the rectal. The passage of 5 oz. of urine over the bulb of the thermometer gives the correct reading in the majority of cases, but about once in every 20 observations a discrepancy occurs which the authors are unable to explain. The time required to obtain a correct reading in the axillary and inguinal regions is usually from 15 to 60 minutes. The authors therefore used the rectal temperature as their guide in determining the effects of exercise and rest. They state that the effect of exercise on the temperature has been generally under-estimated. A very slight amount of movement only is required to produce an appreciable rise of the mercury, while with more exertion they have noted a rise of as much as three and a half degrees. For the purposes of observation three conditions are recognised: (1) Absolute rest. In this condition the temperature is actually depressed. (2) Standing without taking active exercise, which tends to raise the temperature slightly. (3) Active exercise, which distinctly raises the temperature. The effect of slight muscular efforts was tested in this way: on waking in the morning the temperature of a healthy individual was taken; he then got out of bed and stood up, but did not walk, and the thermometer readings were taken off. In one case the temperature was 98·2 degrees in bed; after standing up for half an hour the mercury had risen to 99 degrees. The experiment was repeated on eight healthy subjects, and the average rise obtained was 0·3 degree. Passing on to a consideration of the results of more active exercise the following test was applied. In mild weather a moderately hard bicycle ride of half an hour was taken, which caused slight perspiration. The temperature before starting was —rectal 98·8 degrees and buccal 98·3 degrees. At the end of the ride the readings were—rectal 100·4 degrees and buccal 98·4 degrees. Half an hour after the end of the ride the readings were again taken, and were—rectal 99·5 degrees and buccal 99 degrees. The results of these investigations have an important bearing on the treatment of consumptives in sanatoria, where the amount of exercise which they are permitted to take depends to a certain extent on the amount of the increase of temperature produced by the exercise. The authors of the paper show that this increase is a physiological process which occurs in healthy subjects, but that different individuals show marked differences in the extent of the reaction. Patients who have phthisis have this peculiarity, that their temperatures react more readily to muscular activity than is the case with those who are in a good state of health.

[1] Lancet, March 28.

